# The FDA’s New Guideline “Generally Accepted Scientific Knowledge” (GASK): An Opportunity to Expedite the Approval of Biosimilars

**DOI:** 10.3390/ph16111517

**Published:** 2023-10-25

**Authors:** Sarfaraz K. Niazi

**Affiliations:** College of Pharmacy, University of Illinois, Chicago, IL 60612, USA; niazi@niazi.com; Tel.: +1-312-297-0000

**Keywords:** biosimilars, FDA, pharmacodynamic biomarkers, clinical efficacy study (CES), BPCIA, GASK, receptor binding

## Abstract

The US FDA’s new guideline suggests using “Generally Accepted Science Knowledge” (GASK) to develop nonclinical testing protocols for developing drugs and biologicals to remove unnecessary testing. Interpreting acceptable scientific knowledge as a rational approach has motivated the author to suggest substantial changes to the development of biosimilars, as demonstrated in this paper. The FDA can accept these suggestions without requiring any legislative change to the Act that defines such requirements. Suggested here is the waiving of clinical efficacy testing due to its lower sensitivity compared to analytical and functional testing and pharmacokinetic profiling. Also questioned is the need to test pharmacodynamic markers that do not correlate with clinical response and find new biomarkers requiring extensive testing to validate their use. Should the FDA accept these scientifically rational suggestions, it will significantly reduce the time and cost of approving biosimilars without safety or efficacy risk, as justified based on acceptable scientific knowledge and rationality.

## 1. Introduction

Scientific knowledge tested over time is placed above the knowledge that remains doubtful; however, the “general acceptance” differentiates the two. All need not agree upon a fact, but a consensus makes the knowledge more reliable if not valid. A rare display of this philosophical argument has recently come from the FDA when it released a new guideline on 23 May 2023 that recommends making decisions based on generally accepted scientific knowledge, giving it the acronym GASK with full title of “Draft Guidance for Industry, Generally Accepted Scientific Knowledge in Applications for Drug and Biological Products: Nonclinical Information” (May 2023) [1], explaining the FDA’s thinking on how the developers can rely on GASK when developing both chemical and biological drug products. Moreover, the FDA has also defined the term “nonclinical” in the FDA Modernization Act 2.0 [2]: “‘nonclinical test’ means a test conducted in vitro, in silico, or in chemico, or a non-human in vivo test that occurs before or during the clinical trial phase of the investigation of the safety and effectiveness of a drug, and may include animal tests, or non-animal or human biology-based test methods, such as cell-based assays, microphysiological systems, or bioprinted or computer models.” Additionally, the FDA has provided a detailed description of “pharmacodynamic (PD) biomarkers” [3], which will be a major discussion in this paper since it relates to the application of the GASK. Accordingly, “PD biomarkers are indicators of a drug’s pharmacological effect on its target or targets. For example, the target might be a receptor molecule that initiates a complex signaling cascade. Changes in the levels of proteins along the signaling cascade or modifications to them could be considered pharmacodynamic responses. Therefore, these proteins could be considered PD biomarkers and used to help establish biosimilarity”.

While this guidance is focused on nonclinical testing, it is worth noting that when generic drugs and biosimilars are developed, the nonclinical portions of the dossier comprise the most significant submission. The GASK guidance highlights the flexibility in drug and biologic applications to meet specific nonclinical safety requirements but also allows significant scope in cases when GASK is applied to other kinds of regulatory decision-making.

The Biologics Price Competition and Innovation Act (BPCIA) (Subtitle A) of Title VII—Improving Access to Innovate Medical Therapies (H.R. 3590) [4], where Sec. 7002 details “Approval Pathway for Biosimilar Biological Products” has been applied to the licensing of 44 biosimilar products [5] comprising 12 therapeutic proteins out of more than 250 proteins licensed by the FDA [6]. The term “licensing” has its roots in the history of licensing the manufacturing of biological drugs; this category of drugs was introduced in 1902 [7]. It is noteworthy that not all biologicals are eligible as biosimilars. In 2003, the FDA moved some therapeutic biological products from the Center for Biologics Evaluation and Research (CBER) to the Center for Drug Evaluation and Research (CDER) [8].

- Monoclonal antibodies for in vivo use.
- Proteins intended for therapeutic use, including cytokines (e.g., interferons), enzymes (e.g., thrombolytics), and other novel proteins, except for those that are specifically assigned to CBER (e.g., vaccines and blood products). This category includes therapeutic proteins derived from plants, animals, or microorganisms and recombinant versions of these products.
- Non-antigen-specific immunomodulators (e.g., cytokines, growth factors, chemokines, etc.) that are intended to treat disease by inhibiting or modifying a pre-existing immune response; and proteins or peptides intended to act in antigen-specific fashion to treat or prevent autoimmune diseases by inhibiting or modifying pre-existing immune responses.
- Growth factors, cytokines, and monoclonal antibodies intended to mobilize, stimulate, decrease, or otherwise alter the production of cells in vivo. This category includes growth factors, cytokines, and monoclonal antibodies, as well as non-biological agents, administered as mobilizing agents for their direct therapeutic effect on the recipient, as well as growth factors, cytokines, and monoclonal antibodies administered to subsequently harvest the mobilized, stimulated, decreased or otherwise altered cells for use in a human cellular or tissue-based product (HCT/P).

The FDA has issued several guidelines [9], both draft and final, to help developers better understand the FDA’s current thinking on demonstrating biosimilarity, demonstrating no clinically meaningful differences to a reference product [10]. Despite many changes in the approval process, most developers’ development of biosimilars remains out of reach due to several unnecessary approval requirements, which, given the encouragement embedded in the GASK Guidance, can now be applied more appropriately.

## 2. The GASK Application to Biosimilars

The FDA reviews new drug applications (“NDAs”) under Section 505 of the Federal Food, Drug, and Cosmetic Act (“FD&C Act”) and biologics license applications (“BLAs”) under Section 351 of the Public Health Service Act (“PHS Act”). While it is a requirement for all new drug applications (NDAs) to provide evidence of the safety and efficacy of the drug, including comprehensive reports of investigations, certain NDAs submitted under Section 505(b)(2) of the FD&C Act can fulfill this requirement by utilizing data or information that the NDA sponsor does not possess or have the right to reference. This may include relying on the FDA’s determination of the safety and effectiveness of another drug. The FDA has consistently maintained that “standalone applications,” referring to NDAs BLAs (excluding biosimilar BLAs), must encompass all the requisite facts and information to establish the safety and efficacy of the product. To clarify, the FDA has mandated that an NDA submitted under the 505(b)(1) pathway, or a BLA, must only include data and material held by the applicant or for which the applicant possesses the right of reference. However, this requirement has limited exceptions, which are particular to the nature of the application.

Remarkably, the absence of a comparable alternative to the 505(b)(2) NDA pathway is evident for a BLA candidate. Specifically, the utilization of the FDA’s determination of the safety and efficacy of different medications or biologics is unavailable in this context. It is also not found in the published literature. Suppose a potential applicant for an NDA wishes to utilize the Guideline for the Format and Content of the Clinical and Statistical Sections of an Application. In that case, it is worth noting that even if the FDA ultimately holds a different perspective, the applicant can often utilize this information within a 505(b)(2) NDA. However, a potential BLA program applicant must allocate the appropriate time and resources toward compiling all the requisite material to substantiate their approval or acquire all pertinent references. The GASK Guidance elucidates the FDA’s perspective on the exemption of GASK from the policy. Undertaking this action brings attention to the inflexibility of the overarching policy—and potentially reveals a path for a more lenient implementation of this regulation.

The GASK Guidance refers to medical or scientific information widely recognized by professionals with scientific expertise and experience in the relevant field, including experts from the FDA. It primarily encompasses long-standing scientific concepts that have gained widespread acceptance. The proposed advice explains that GASK may also rely on a substantial body of scientific research or material deemed to have broader applicability beyond the specific contexts in which it was initially generated. The draft guidance does not include a specific definition for the term “sufficiently large”. The intended scope of this term by the FDA could encompass a range of possibilities, including a small number of studies, a moderate number of studies, or even a substantial number of studies. Likewise, the acronym GASK could potentially originate from “textbook excerpts containing fundamental scientific principles” when “particular products are not explicitly referenced as the origin of the information”.

The goal of the GASK Guidance is to intentionally avoid providing a clear distinction between the published literature, which is not allowed in a standalone application, and GASK, which is allowable in a standalone application. However, the guidance outlines two scenarios in which it may be suitable to utilize GASK to fulfill nonclinical safety criteria.

Sometimes, using GASK may be an alternative to conducting specific nonclinical safety studies on substances commonly found in a healthy human body. These substances encompass endogenous and exogenous compounds present in the diet, excluding dietary supplements. This substitution applies only when the drug is an unaltered substance administered orally. The degree of exposure is equivalent to that of the endogenous drug, or in the case of an exogenous substance, the exposure level does not exceed customary limits of dietary intake.

Furthermore, the GASK can be employed as an alternative approach to elucidate the effects of a modified biological mechanism or route, obviating the need for targeted pharmacology and/or toxicology investigations to quantify the pathway’s impact. The utilization of GASK by the FDA is restricted to products that exhibit notable impacts on established biological pathways, usually in cases where the drug-induced modification of a pathway leads to unfavorable consequences. Consequently, while this example may have limited applicability in the context of novel drug development, it does present novel prospects for creating biosimilars.

The FDA’s confirmation that it will allow reliance on GASK in presenting testing protocols should signal an opportunity for the broader use of scientifically justified information in novel standalone applications. While the advisory footnote explicitly states that it cannot be applied to other circumstances, such as clinical investigations, the draft guidance acknowledges the potential benefits of relying on data and information rather than conducting studies, which include streamlining development processes, reducing associated costs, expediting a drug’s approval and marketing, and ultimately making it more readily accessible to patients. The statement acknowledges the potential appropriateness of utilizing external data and information in addition to a standalone application. Still, it fails to provide a legal or policy justification for restricting the scope of the draft guidelines to nonclinical data.

While the principle of relying on scientific evidence forms the basis of regulatory guidance, it has not been explicit in the past as to what is “generally accepted,” what is “scientific,” and what “knowledge” is relevant to deciding on the use of prior knowledge. This guidance brings clarity by first explaining that it applies to two types of substances: one that occurs naturally in the body (e.g., therapeutic proteins) and where its effects or mechanisms of action are known, and the second where the impact of the drug is known in the context of a particular pathway (e.g., receptor binding, in the case of monoclonal antibodies) or where a drug has an on- or off-target status that can impact the biological path or demonstrates a known molecular mechanism of action that leads to pharmacological and toxicological outcomes; all of these apply to monoclonal antibodies. While these newer suggestions by the FDA will take time to be understood well, the fact that the FDA is already proposing to reduce testing should give validity that proper scientific arguments will change the mindset of regulatory authorities.

The GASK Guidance advises sponsors who wish to utilize GASK to promptly submit their justification to the appropriate review division during the product development phase to receive input regarding the planned regulatory approach for authorization. When applied to the development of biosimilars, the guidance provides many opportunities, whether identified in the guidance or not, to bring these issues to the attention of the FDA. This prospect is anticipated and suggested in this paper.

## 3. Regulatory History

The initial guidelines of the FDA, according to the BPCIA, were an extension of the guidelines applied to new biologicals. However, their safety and efficacy were confirmed as biosimilars entered the market. This led the FDA and other regulatory agencies to revise these guidelines based strictly on scientific considerations, a practice now formally described in the GASK Guidance. The term “scientific” should be interpreted broadly, meaning what is rational and well-proven. Given below is a historical summary of the changes in the FDA guidelines and perspective relating to biosimilars:

- March 2010: The BPCIA of 2009 was signed into law, creating a new class of biological drugs, namely biosimilars. It was amended to extend the exclusivity to twelve years from the initial eight years at the time of the approval of BPCIA. This change came at the protest of “big pharma,” claiming that the cost of developing new biological drugs has reached billion-dollar ranges. That extended times were needed to recover their investments. The EU allows exclusivity of ten years. In 2022, a new law, the Inflation Reduction ACT (IRA) [11], allowed the CMS (the agency responsible for Medicare and Medicaid patients) to reduce the price of biological drugs by 35% if they had a monopoly of 12 years, if there are no biosimilars and none expected within 12 months, and if these drugs are among the top ten highest reimbursed drugs. Surprisingly, the associations responsible for promoting biosimilars also opposed this bill, stating that reducing the price of reference products would reduce the incentive for biosimilar development efforts.
- February 2012: The FDA released draft guidance on biosimilar product development, outlining the scientific and regulatory considerations for demonstrating biosimilarity to a reference product.
- August 2014: Reference Product Exclusivity for Biological Products; Draft Guidance for Industry.
- April 2015: Scientific Considerations in Demonstrating Biosimilarity to a Reference Product; Guidance for Industry.
- April 2015: Quality Considerations in Demonstrating Biosimilarity of a Therapeutic Protein Product to a Reference Product; Guidance for Industry.
- December 2016: Clinical Pharmacology Data to Support a Demonstration of Biosimilarity to a Reference Product; Guidance for Industry.
- January 2017: The FDA issued final guidance on interchangeability, providing recommendations on demonstrating that a biosimilar can be substituted for its reference product without the involvement of the prescribing healthcare provider.
- March 2018: The FDA released a Biosimilar Action Plan outlining the FDA’s commitment to promoting competition and access to biosimilars through various initiatives, including educational outreach, regulatory clarity, and market competition.
- May 2018: The Citizen Petition by the author to the FDA asked for a comprehensive review of the biosimilar guidelines to bring more rationality and scientific understanding of the testing required to approve biosimilars [12]. The FDA responded six months after the filing on 18 November 2018 [13], stating that it needed more time to review the requests made in the Petition, even though a reply is expected within 120–180 days [14]. The FDA has adopted several suggestions in the Petition:
- This Petition suggested that the FDA adopt scientific principles to conclude the arguments for removing animal toxicology testing. The BPCIA has been amended as of the end of 2022, removing the term “animal toxicology” and replacing it with “nonclinical testing” [15],
- The Petition had suggested removing the flawed statistical testing models. The FDA withdrew pivotal guidance, i.e., Statistical Approaches to Evaluate Analytical Similarity [16,17,18], and issued a new guideline [19] in May 2019: Development of Therapeutic Protein Biosimilars: Comparative Analytical Assessment and Other Quality-Related Considerations Guidance for Industry. This guideline removed the tier 1 testing that was challenged in the Citizen Petition as non-scientific [20]. The statistical issue related to testing critical quality attributes, such as protein content and potency, requires the 90% confidence interval of biosimilar attributes to fall within 1.5 times the standard deviation in the reference product. The contradiction occurred because these attributes also meet the release specification that is fixed and different.
- The FDA adopted several suggestions in the Petition, including beginning an extensive teaching program about biosimilars [21] for all stakeholders. This action by the FDA has brought about significant change in the perception of the safety of biosimilars. Most recently, the FDA has changed the labeling requirements of interchangeable biosimilars to remove them from being listed as an interchangeable biosimilar, proposing that the interchangeable status is a legal designation, not related to quality matters; this change will significantly impact the perception of the safety and efficacy of the two classes of biosimilars in the US, a situation that needs a change, as discussed below.
- The denied recommendations included removing the four-letter suffix system to identify biological products, not just biologicals. An argument that this differentiation is needed to monitor post-market data is irrelevant since the registration numbers assigned to products are sufficient to meet this requirement. However, having gone this far, it is unlikely that there will be any change concerning this ruling by the FDA, and perhaps it is unnecessary to question it.
- Other recommendations to be considered include allowing approved non-US comparators to use essentially the same dossier as the US-licensed products and to declare to the public that biosimilars have “no clinically meaningful difference” from the originator product.
- The final recommendations are to encourage the development of in vitro immunogenicity testing methods to reduce test subject exposure on ethical grounds, to revise the perspective on the clinical relevance of the protocols and statistical methods used to establish PK/PD similarity, to end clinical efficacy testing in patients, and to remove the interchangeable status of biosimilars. In vitro immunogenicity tests assess the potential of a biological drug to induce an immune response in patients. Common techniques include enzyme-linked immunosorbent assays (ELISAs) [22] and cell-based assays [23] that measure the presence of anti-drug antibodies (ADAs) and neutralizing antibodies (NAbs). Furthermore, cell-based assays like the competitive ligand-binding assay (CLBA) are employed to assess the impact of ADAs on a drug’s efficacy. One notable example is the anti-TNF (tumor necrosis factor) biological drug infliximab [24]. Another drug that has undergone in vitro immunogenicity testing is adalimumab [25], and another is anti-TNF biologic [26]. Furthermore, in the context of cytokines, interferon-beta [27] and erythropoietin [28] have also been subjects of in vitro immunogenicity studies.
- May 2018: The FDA issued a letter to the United States Pharmacopoeia (USP) asking it not to create monographs for biological drugs [29]. “Because USP’s proposed revisions would aggravate existing concerns that a monograph could impede or delay the licensure of biosimilars and other biological products, the FDA strongly encourages USP to withdraw its proposal. The FDA welcomes future interaction with USP on these issues to ensure that biological product monographs do not create an unnecessary barrier to the availability of biosimilars and other biological products to patients. For example, we see opportunities for optional methodological standards that could encourage innovation and product development”. One of the FDA’s concerns was that using the analytical methodologies provided by the originator product companies might result in complex intellectual property issues. The USP has dropped its plan to create monographs of biological drugs.
- June 2018: The FDA withdraws Draft Guidance for Industry: Statistical Approaches to Evaluate Analytical Similarity, as recommended in the May 2018 Citizen Petition, as it required quality attribute comparisons based on a 90% confidence limit within 1.5× standard deviation of the reference product. This analysis conflicted with the release specification allowance and presented no scientific basis. The FDA also switched from “comparison” to “assessment” to give the evaluation a broader meaning.
- May 2019: The FDA issues Considerations in Demonstrating Interchangeability with a Reference Product; Guidance for Industry, a final guideline. The FDA can allow interchangeability without requiring the proposed three switching and alternating studies with the reference product to secure interchangeable status. The FDA cannot remove the interchangeable classification, unlike the May 2018 Citizen Petition. In February 2020, the FDA added clarification for allowing fewer conditions than the reference product in case it is dictated by the intellectual property or the choice of biosimilar developer. In 2023, the FDA issued a new guideline on labeling biosimilars, removing the mention of the interchangeable status of biosimilar in the prescribing information, asserting that this status is a legal description, not a quality classification.
- July 2021: The FDA released a Biosimilars Action Plan Progress Report, providing an update on the FDA’s efforts to enhance the biosimilars market and increase competition. The plan includes in silico testing and many other AI/ML-based technologies that biosimilar developers could adopt. The FDA issued a Biosimilars Action Plan [30] that encourages using in silico methods; the FDA has also removed the requirement for the immunogenic testing of products that have not impacted pharmacokinetics, such as insulin, and will allow the same for other products.
- December 2022: The FDA Modernization Act 2 amends the BPCIA, removing the term “animal toxicology” and replacing it with “nonclinical” after scientific evidence was presented against animal testing [31]. The scientific basis of this amendment came from the understanding that animals may not have the receptors required for biological drugs to act; the receptor binding leads to PD, leading to pharmacology that results in clinical and toxicological properties. This amendment required legislative action since the term “animal toxicology” was embedded in the requirement for biosimilars.
- December 2022: the FDA publishes its recommendations on using pharmacodynamic markers to support the efficacy of biosimilars [32], as well as a pivotal paper in January 2023 to establish the role of PD markers [33] in the waiving of clinical efficacy testing. The FDA conducted clinical trials to “discover” PD biomarkers, but these studies were limited to proteomics testing and did not differentiate between a PD biomarker and a pharmacology biomarker. The FDA also concluded that the PD biomarkers used to gain a waiver for clinical efficacy testing need not correlate with clinical efficacy, a weak point in justifying the use of PD biomarkers.
- November 2022 and July 2023: The US Senate received a bill to remove the interchangeability status of biosimilars based on scientific evidence that such studies can never fail and thus are tantamount to human abuse. This bill is opposed by the originator companies and even some biosimilar companies that have the additional resources to secure the interchangeable status to help them promote their product as superior to another biosimilar that does not have this status.
- August 2023: The CMS makes its first choice regarding drugs approved for price reduction [34], including three therapeutic proteins, Stelara (ustekinumab), Enbrel (etanercept), and FlaspPen and Novolog Pen (insulin aspart), because there were no biosimilars approved for these drugs; however, it is anticipated that by the time this selection becomes effective, the licensing of biosimilar will likely remove them from the list [35].
- September 2023: A Citizen Petition filed by the author suggests that the FDA consolidate its biosimilar guidance to follow the advice given in the May 2018 Citizen Petition by the author, with updates on how to approve efficacy testing waivers and other regulatory changes within the power of the FDA. This Petition identified the changes made by the FDA as suggested in the May 2018 petition and recommended additional recommendations that are detailed later in this paper.
- September 2023: The FDA calls a meeting to understand the scientific evidence for removing the clinical efficacy testing of biosimilars [36]. The MHRA (the UK Regulatory Agency) declared that it no longer requires patient efficacy testing based on a lack of scientific validity.
- September 2023: The FDA issues a new guideline, namely Labeling for Biosimilar and Interchangeable Biosimilar Products [37], allowing the removal of identification if it is approved as an interchangeable biosimilar, stating that the interchangeable status is a legal description and not a quality differentiation, and thus stakeholders do not need this information, leaving it available only in the Purple Book.

While the FDA has come a long way in promoting science in establishing biosimilarity, there remains a significant unmet need to extend the concept, as presented in this paper, to bring greater rationality to the reduction in the cost of development, as well as the removal of unnecessary human testing in concordance with the Helsinki Agreement. This paper lists the changes and scientific arguments to support the thesis, aiming to ensure that biosimilars are safe and effective yet developed at a cost that will make them affordable.

## 4. Patent Dance

The litigation of patents for biosimilars presents a distinctive circumstance. The commencement of a patent dance is initiated promptly upon the submission of a biosimilar application. To initiate the patent dance, the applicant seeking approval for a biosimilar product submits its Biologics License Application (BLA) for the biosimilar to the sponsor of the reference product. Additionally, the applicant provides supplementary details about its manufacturing method. The reference product sponsor must furnish the biosimilar applicant with a compilation of unexpired patents within 60 days of this initial interaction, for which a plausible assertion of infringement could be made. The applicant seeking approval for a biosimilar product is given 60 days to present their perspectives on noninfringement and invalidity. The reference product sponsor is granted 60 days to deliver their response and positions. The sponsor of the reference product must promptly update its list to include any recently granted patents within 30 days from the date of issuance of said patents. After the early rounds of disclosures, the involved parties proceed with a sequence of responses and negotiations, which may ultimately lead to the reference product sponsor initiating legal action for patent infringement in a United States federal court. It is worth noting that in 2017, the Supreme Court decided that the patent dance was not obligatory, as it lacked enforceability through injunction action [38].

The timely availability of patent information about licensed biologic products has significant value for biosimilar applicants. The limited duration of the patent dance confers a strategic benefit to a biosimilar applicant, as it is advantageous to acquire a comprehensive understanding of the patents disclosed by the reference product sponsor before the commencement of the dance. This information will allow a biosimilar applicant to formulate noninfringement and invalidity arguments. On the contrary, if a sponsor of a reference product possesses robust patent protection, an applicant seeking to develop a biosimilar may opt to delay seeking permission until the reference product sponsor’s patents expire. By adopting this approach, the reference product sponsor can effectively circumvent the expenses of participating in the patent dance and subsequent legal disputes.

The patent dance remains one of the most significant hurdles in approving biosimilars, both for the delays in bringing approved products to market and the costs of these lawsuits. The 37 lawsuits have resulted in a cost of over a billion dollars to biosimilar developers and significant delays in the entry of biosimilars. Table 1 lists the status of these lawsuits.

The US Congress has passed 25 biosimilars-related bills [40] out of 234 introduced [41]. One hundred bills have addressed patents approving biosimilars [42], and eighteen have become law [43]. In 2019, nine bipartisan senators introduced the Biologic Patent Transparency Act to require biologic makers to disclose their patents publicly [44]. In December 2020, Congress passed a large appropriations bill with a section (Section 325) titled “Biological product patent transparency” [45].

The inclusion of patent information about biologics and FDA regulatory exclusivity information in the “Purple Book” database was a direct consequence of the Purple Book Continuity Act (PBCA) implemented by the FDA [46]. The enhanced database will offer novel prospects for industry professionals to identify (i) prospective partnerships for the licensing of current intellectual property, (ii) fresh strategies for the enforcement and safeguarding of intellectual property, and, potentially, (iii) avenues for the advancement of new technology.

As stipulated by the Act, it is required that each reference product sponsor involved in a patent dance must furnish the FDA with a comprehensive inventory of patents that have been disclosed to a biosimilar applicant as part of the patent dance process. The provision of information on patent expiry must be made within 30 days from the time of disclosure to the biosimilar applicant. The FDA must incorporate the patent and expiration details of the biologic in the Purple Book. Including exclusivity information in the Purple Book is contingent upon the biologic or biosimilar meeting the criteria for exclusivity. However, it is essential to note that not all exclusivity periods are required to be identified in the Purple Book.

One notable distinction between the recently introduced Purple Book standards and the content included in the preexisting Purple Book is in the incorporation of patent information. Previously, information between a reference product sponsor and a biosimilar applicant during a patent dance was considered confidential. Including patent information in the Purple Book significantly impacts the extent and availability of patent-related information about approved biological products.

Nevertheless, it is essential to acknowledge that the Purple Book has certain limitations regarding its information. Including supplementary patent information does not impact the initial biosimilar that applies after deploying the modifications to the Purple Book. The reference product sponsor’s identification of patents during the patent dance may remain undisclosed, leaving others uninformed. Before submitting a biosimilar application, patent information would not be available in the FDA database. Furthermore, the legislation fails to address the potential consequences if the biosimilar sponsor chooses not to participate in the patent dance.

In contrast to the Orange Book, the Purple Book does not require product sponsors to consistently update patent information, except during the patent dance process. The need for a product sponsor to update information only arises during a patent dance with a biosimilar. Consequently, future applicants should refrain from only depending on the Purple Book as their primary resource for identifying and comprehending the prospective patent landscape.

However, the inclusion of patent information in the Purple Book will facilitate the ability of the following applicants to prepare noninfringement more effectively and invalidity positions before engaging in the patent dance. The provision of advance notice can play a crucial role in the legal and business plans of a biosimilar applicant seeking to file for a biosimilar. Furthermore, disseminating information can also serve as a valuable means to acknowledge product sponsors. For instance, when a sponsor of a reference product possesses robust patent protection documented in the Purple Book, it can discourage a biosimilar applicant from pursuing approval before the expiration of the sponsor’s patents.

According to the recently enacted regulation, it is a requirement for the Purple Book to specify the duration of exclusivity periods for both unexpired reference products and interchangeable products. It is worth noting that according to the language of the Act, the specified periods of exclusivity are mandated to encompass solely the 12-year exclusivity for reference goods and the exclusivity granted to interchangeable biosimilar products. Although the data download provided by the Purple Book does contain information regarding orphan exclusivity, companies should consult the FDA’s orphan drug database to verify the presence of unexpired orphan exclusivity periods for a given product. Including six-month pediatric exclusivity extensions in the Purple Book exclusivity periods remains uncertain.

Furthermore, it should be noted that the regulation stipulates that posting exclusivities is only mandatory in cases where the FDA has officially determined the eligibility for exclusivity. In the context of small molecules, it is common for the FDA to delay making official conclusions regarding exclusivity until the matter reaches a suitable stage, such as when an abbreviated new drug application or a 505(b)(2) new drug application is submitted. Suppose the Purple Book adheres to the same principles as the Orange Book. In that case, not all newly permitted biologics may have exclusivity periods, as the FDA may not have officially determined exclusivity in every instance. It is worth mentioning that the most recent data downloaded from the FDA’s Purple Book include reference product exclusivity periods for certain listed items while omitting this information for others.

Furthermore, using patent information from the Purple Book in conjunction with the patent dance enables enterprises operating in the biologics sector to identify novel business prospects through various means. Initially, biotechnology businesses can examine the patent details provided in the Purple Book while considering their pre-existing technological capabilities. Organizations can utilize the gathered data to discern prospective licensing or cooperation prospects with other sponsors of reference products or with a biosimilar application. Suppose a reference product sponsor lacks patent protection for a particular aspect of its manufacturing process. In that case, it may express interest in acquiring or leasing patents that another company covers in the relevant area. The reference product sponsor would derive an advantage from this proposal as it would enhance the level of patent protection for their certified product. Suppose a reference product sponsor possesses patent protection within a specific domain. In that case, it is plausible for another company to possess technological capabilities that enable a biosimilar applicant to circumvent said protection. In such cases, prospective biosimilar applicants may express interest in collaborative efforts about said technology.

Furthermore, an organization has the potential to utilize the patent information provided in the Purple Book to effectively commercialize intellectual property that bears resemblance to or intersects with preexisting patents. In some instances, a reference product sponsor may exhibit interest in acquiring the rights to intellectual property, even though the sponsor already possesses patent protection within the corresponding domain. This interest may be particularly evident when the intellectual property predates the sponsor’s existing patents. The reference product sponsor can gain enhanced patent protection for its product because of this development. However, a biosimilar applicant may express interest in collaborating to initiate a legal procedure at the patent office to render a reference product sponsor’s patent invalid. This endeavor would rely on using another company’s intellectual property as the foundation for the invalidation process.

In conclusion, while evaluating the patent information in the Purple Book, a corporation must deliberate on potential avenues for future technical advancements. Suppose a corporation possesses specialized knowledge in a particular technological domain. In that case, another company may have the opportunity to leverage that experience for collaborative purposes with a reference product sponsor or a biosimilar applicant in the pursuit of developing novel technologies.

Ideally, the patent dance should be removed, but this would require legislative action that is highly sought after in the US Congress. Still, the forces of the originator companies have successfully prevented this from happening.

## 5. Bridging Studies

The expenditure of developing biosimilars is substantial, necessitating developers to devise a comprehensive worldwide approach using a single regulatory dossier to obtain regulatory approvals across numerous jurisdictions. The BPCIA mandates that a biosimilar must demonstrate similarity to the US-licensed originator, which refers to a product approved under Section 351 (a) of the Public Health Service Act of 1942, as amended. Consequently, developers must conduct three-way studies involving a US-licensed product, a non-US product, and a biosimilar candidate to compile their regulatory dossier.

However, the law explicitly grants the FDA the authority to exercise discretion in determining the necessary information for establishing biosimilarity, as outlined in Section 42 USC 262(k)(2)(A)(ii). The BPCIA refers to the reference product as a US-licensed product. Still, the FDA can consider a non-US product as US-licensed only if registered using the same dossier (except for administrative differences). This acceptance by the FDA will significantly enhance the entry of biosimilars without the additional bridging study, as currently required. The FDA should invoke its authority to accept non-US reference products if these are approved in another jurisdiction using essentially the same registration dossier used in the licensing of the US product [47]. This is a critical step to enable the global registration of biosimilars.

The policies on bridging studies range from no bridging studies to repeated PK/PD studies. The FDA has the authority to waive bridging studies under Section 42 USC 262(k)(2)(A)(ii) if the proposed reference product meets all the composition, indication, and route of administration requirements and is approved using “essentially” similar regulatory dossiers.

## 6. Analytical Assessment

**Figure 1:**
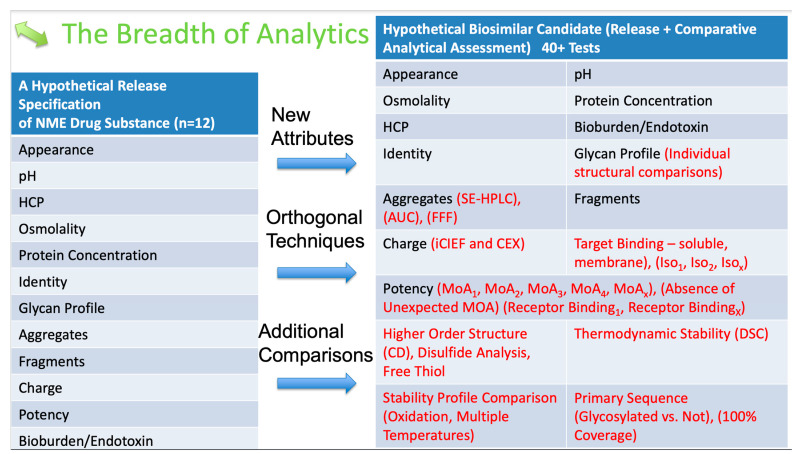
FDA-proposed analytics for product and process-related critical quality attributes. Test methods in red are the newer FDA recommendations.

Analytical assessment is the most decisive proof of biosimilarity, particularly with advanced analytics that allow orthogonal comparisons. The FDA has removed its initial guideline that prescribed tier 1 testing for critical quality attributes and suggested the two most essential attributes: protein content and potency. The developers are also required to analyze and find the CQAs. While the FDA guidance does not specify the testing attributes, the FDA has broadly made such suggestions (Figure 1) [48].

There are two quality attributes: one related to the product dependent on the expression system relatively invariable and the other to the process that can be tailored to match the reference product within limitations. The product-related attributes are critical, while the process-related attributes are part of release specification, such as the protein content and potency that should not be made part of the analytical assessment exercise, as assumed by most developers and the FDA. The release specifications that are scientifically justified for injectable products should be based on legacy attributes such as protein content, potency, particle size, subvisible particles, sterility, etc. Additionally, the specifications of the process-related attributes, such as post-translational modification, impurities, and other critical attributes, are established by testing multiple fields of the reference product.

### 6.1. US Pharmacopoeia

One solution to reduce the burden of analytical testing is to allow the US Pharmacopoeia to develop monographs for biological drugs as it has done for drugs like insulin. The FDA has clashed with the USP [49] based on the conclusion that biologics makers can use the monograph process to block biosimilar competition by incorporating patented characteristics of their product irrelevant to safety, purity, or potency, further impacting competition. However, this is a misconception since the USP can practice the profile development as a biosimilar developer without relying on the reference product company to share data. Having validated test methods and product specifications will significantly lower the barrier to the entry of biosimilars. Suppose the FDA agrees to accept compliance with the monographs as proof of biosimilarity. This would dramatically reduce this critical step’s testing burden, time, and cost in developing biosimilars.

One scientific argument within the GASK approach relates to the Guideline Q5E [50] that applies when a manufacturing change is brought in a biological product. This is a more straightforward protocol wherein the newer site product is compared with the older product on limited analytics; an analogy is drawn here with the USP testing of the reference product that can be considered a pre-Q5E product. Another area of concern that the FDA needs to remove is the USP specification testing; for example, insulin is tested via the chromatography method in all compendia, including the USP, but the FDA still requires obsolete rabbit testing [51]. There is a need for the FDA to allow developers to present their plan for compliance with the reference product, using the fewest number of test batches, particularly for the product-related attributes. Comparative testing using statistical models requires a more significant number of lots, which may not be necessary for the attributes that do not vary much. The goal of the analytical assessment should be to ensure attribute variation no more than the reference product. The USP can conduct this exercise using multiple batches, and the developers should be allowed to use these specifications, especially since the test methods used will also be drawn from the USP monographs.

The current expectations of the FDA involve using 8–10 lots of the reference product and a similar number of the biosimilar candidate, an exercise that costs millions of dollars; a USP-monograph would eliminate most of this cost without compromising safety and efficacy. The USP could also develop test methods to reduce development costs and intellectual property risks.

### 6.2. Immunogenicity Study

Immunogenicity is anticipated for proteins; if the antidrug antibodies produced do not alter the pharmacokinetic profile, such studies are no longer required [52]. Immunogenicity assessment has a role in demonstrating product comparability following manufacturing changes and similarities in the context of biosimilar development. Even minor changes can potentially affect the bioactivity, efficacy, or safety, including the immunogenicity of a therapeutic biologic. All these attributes are tested under the Q5E compliance when a change is made [53]. However, suppose the immunogenicity relating to the generation of anti-drug antibodies does not impact the disposition profile. These differences are not considered relevant, as listed in the FDA guidance on insulin immunogenicity testing [53].

The FDA is developing this complex science of predicting immunogenicity using in vitro methods [54], promoting vitro immunogenicity assays.

In vitro, immunogenicity testing is essential for evaluating the immune response elicited by biopharmaceuticals, including vaccines and therapeutic proteins. One approach involves antigen presentation assays, which assess a biologic’s processability and presentation via antigen-presenting cells [55]. T-cell proliferation assays are another critical aspect, gauging T-cell activation and proliferation when exposed to the biological [56]. Furthermore, cytokine release assays measure the extent of immune cell activation by monitoring cytokine release [57]. B-cell epitope mapping, identifying the specific biologic regions recognized by B-cells, also plays a crucial role [58]. Predictive models utilizing computational tools to forecast biologics’ immunogenicity [59] and comprehensive assay development for accurate testing are equally important [60]. These in vitro tests, though invaluable, often require complementation by in vivo studies to offer a well-rounded insight into biopharmaceutical immunogenicity.

There is an ethical risk in testing for immunogenicity in healthy subjects, as we can make them immune-positive, and testing in patients may not be sensitive enough due to compromised immune systems. For the FDA to move the science of in vitro immunogenicity testing further, the FDA should:

- Let developers present in vitro tests instead of clinical immunogenicity testing where required.
- Continue its internal development in finding and prescribing testing modalities that reduce the need for clinical immunogenicity testing.

## 7. Clinical Pharmacology

While the FDA has encouraged developers to present novel testing protocols for PK/PD studies, the FDA should bring guidance to expand the utility of these studies. For example, the receptor binding simulation can be made by reporting the change in the distribution volume as a function of time, conducting multi-dose studies and parallel design studies to reduce the study cost while also avoiding repeat exposure to healthy subjects that may result in immunogenic responses putting the study subjects at a higher risk.

## 8. Clinical Efficacy Studies (CES)

The BPCIA states:

“(cc) a clinical study or studies (including the assessment of immunogenicity and pharmacokinetics or pharmacodynamics) that are sufficient to demonstrate safety, purity, and potency in 1 or more appropriate conditions of use for which the reference product is licensed and intended to be used and for which licensure is sought for the biological product.”

This clause presents many contradictions and misconceptions that can be resolved, invoking the GASK applications.

The provision that testing in just one indication may be sufficient to demonstrate clinical equivalence is fallacious since the modes of action can differ among the approved indications. A recent study has confirmed that antibodies employ different modes of action, and these differences cannot be identified through in vitro studies [61]. Testing in only one indication cannot remove the uncertainty about the safety or efficacy of a biosimilar candidate. Leaving these required studies in one indication makes this merely a checklist item.

**Figure 2:**
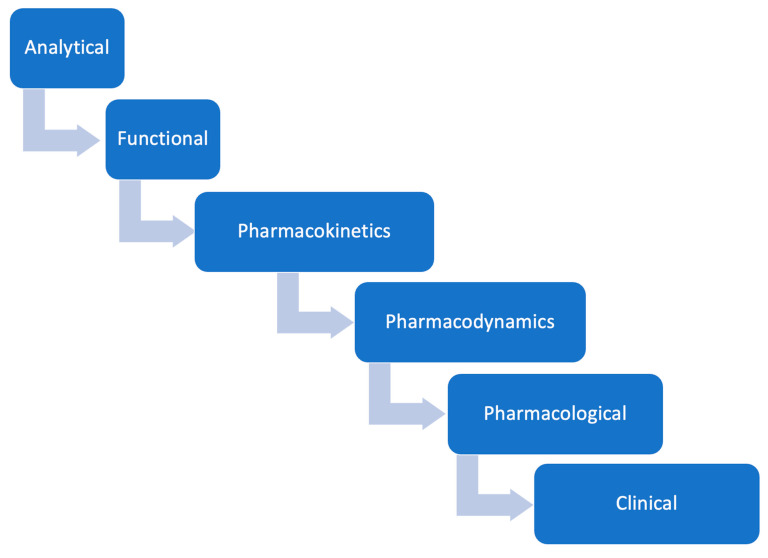
Drug testing steps from most reliable to least reliable when comparing attributes of two products.

An assumption in the legislation is that clinical efficacy testing is the last step of establishing safety and efficacy; the fact is that comparative clinical efficacy testing is the least sensitive of the other required testing procedures, i.e., analytical assessment and clinical pharmacology profiling (pharmacokinetics, pharmacodynamics, immunogenicity) (Figure 2) [62].

Historically, products have been approved even when they failed the clinical efficacy testing, but never if there were any variations in prior analytical and clinical pharmacology testing. In one instance, a trastuzumab clinical study failed in a double-blind active-control protocol where the primary efficacy endpoint was the complete response rate of differences in pathological CR rates; however, an analysis conducted that excluded patients given an ADCC variable reference product showed that the pre-specified equivalence margin was met [63].

The inevitable weakness in the study design includes using clinical judgment to establish an acceptable difference and study size that cannot be calculated with confidence due to large inter and intra-subject variability and small anticipated differences that require a very large patient population to make any study meaningful. As a result, almost none of the studies have failed. Even when a CES fails to meet the equivalence criteria, these are approved, such as trastuzumab, where excluding subjects with high PPD normalized the results.

The inability to conclude whether a study has failed is based on Bayesian calculations that show the probability closer to zero based on posterior probability. Comparative efficacy testing has not led to any product withdrawals or recalls from the market. These data are available in the 108 EPAR files from EMA [64] and 42 approval documents from the FDA [65]. No failed studies were reported when conducted in a comparator mode; in a couple of cases, the acceptance criteria were revised retroactively to meet the equivalence. The studies reported on the clinicaltrials.gov portal [66] show that over 200 studies for which the results are reported met the acceptance criteria. In addition, the PubMed database lists 504 randomized control clinical trials that showed no clinically meaningful difference [67].

Clinical efficacy testing of new drugs against a placebo is a gold standard that has recently been criticized. Dr. Janet Woodcock of the FDA stated: “Why should we put patients through all these different trials just to check a box.” The FDA has recently questioned this idea of real-time testing, claiming that clinical efficacy testing is “broken” [68]. and that, following the 21st Century Cure Act, new digital technologies and real-world evidence (RWE) are necessary [69,70].

Despite the data demonstrating that the efficacy testing of biosimilars is redundant, the FDA has yet to acknowledge this. Therefore, the text in the BPCIA should be corrected as follows:

“(cc) a clinical study or studies (including the assessment of immunogenicity and pharmacokinetics or pharmacodynamics) that are sufficient to demonstrate safety purity.”

However, a change in the language of the BPCIA requires a legislative change, but the BPCIA has given the FDA the right to make this change. Based on GASK considerations, it will be appropriate for the FDA to declare, as was recently done by the MHRA, that no CES is required.

### 8.1. CES Alternatives

While the MHRA has declared that no CES should be conducted [71], and all other regulatory agencies have shown interest in letting the developers present arguments for the waiver of the CES [72], there is a general tendency toward replacing CES with another testing instead of removing it as the MHRA has done. The FDA has taken the conservative approach of removing efficacy testing for drugs with known pharmacodynamic biomarkers that are linearly related to clinical response, such as erythropoietin, granulocyte stimulating factor, and other cytokines.

Many biological drugs, such as antibodies, do not display such linearly related PD biomarkers. Yet, the FDA recommends that any PD biomarker, whether it is related to clinical response or not, can be used for comparison purposes. A direct quote from the FDA states: “PD biomarker use in biosimilar development is meant to demonstrate similarity rather than to independently establish the safety and effectiveness of a biosimilar product, so considerations for PD biomarkers intended to support a demonstration of biosimilarity are different from considerations to support new drug approvals. As such, a correlation between the PD biomarker and clinical outcomes, while beneficial, is not necessary” [72]. This statement creates a logical problem. While the purpose of using a PD biomarker is to demonstrate similarity, there can never be an assurance of linearity within the distribution of the PD biomarker and the clinical response. If the PD biomarker test meets the similarity test, it cannot ensure clinical similarity. If it does not match, it also does not mean that the two products are not clinically similar. Based on the GASK principles, this conclusion nullifies the utility of PD biomarkers.

Despite the conclusion that PD markers have little value, the FDA conducted applied research on PD biomarkers to facilitate biosimilar development [73]. This research included clinical pharmacology studies in which participants receive varying biological doses of a drug, and investigators discovered new biomarkers. To accomplish this goal, the FDA suggests using omics technologies to find new biomarkers [74], an unnecessary and impractical exercise for biosimilar developers. The clear disadvantages of these suggestions include the following:

- The analysis and validation of biomarkers will require extensive research, which should not be expected of biosimilar developers.
- The cost of identifying and validating a biomarker may exceed the CES, making it an improbable alternative.
- Biosimilars use different biosimilars; some may be easier to match than others.
- The lack of correlation between a biomarker profile and clinical response leads to uncertainty about whether meeting the biomarker profile is clinically meaningful.
- The nonlinearity of the biomarker profile cannot be established; thus, it will be impossible to conclude whether a failed matching is a study failure, and the same will be true if a study passes.

### 8.2. Known Biomarkers

The FDA statement that biomarkers need not correlate with clinical response categorically makes this exercise irrelevant. However, there is a likelihood that the FDA may disagree with this argument and still suggest using PD biomarker comparisons. In such cases, options are available other than discovering new biomarkers. When a new biological product is approved, its PD properties are generally reported and can be used to satisfy the regulatory requirements despite being a checklist item. Examples of these PD biomarkers are presented in Table 2. However, validating any marker, whether already reported or newly discovered, often remains insurmountable, a fact that the FDA should recognize. This inevitable nature of testing further leaves such testing as merely a checklist item.

### 8.3. Receptor Binding

In the cascade of events, before a PD response is triggered, the protein molecule first binds to its receptors, a well-known and established mechanism of action (Figure 2).

The clinical response to many biological drugs is triggered by first binding to a receptor [106] routinely tested in the functional assay platform. Thus, the functional assays provide the most sensitive similarity comparison [107]. Current science has made this testing highly accurate and objective. mAbs can also interact with multiple receptors and can be evaluated using orthogonal analytical methods. The primary receptors involved in the activity of the therapeutic proteins include (parenthetical entry shows the number of such receptors) the following: Glucagon-like peptide 1 (GLP-1) receptors (3), insulin receptors (3), heat-stable enterotoxin receptors (2), Adrenocorticotropic hormone receptor, Angiotensin II type 2 (AT-2) receptor, Corticotropin-releasing factor receptor 1, Glucagon-like peptide 2 receptors, Gonadotropin-releasing hormone receptor, Notch signaling pathway, Oxytocin receptor, Parathyroid hormone receptor, Parathyroid hormone/parathyroid hormone-related peptide receptor, Prothrombin, receptor tyrosine-protein kinase erbB-2, Secretin receptor, Somatostatin receptor 2, Somatostatin receptor 5, type-1 angiotensin II receptor, Vasopressin V1a receptor, Vasopressin V1b receptor, Vasopressin V1a receptor, Vasopressin V1b receptor, and Vasopressin V2 receptor [108].

Since the receptor binding tests are often quantitative, they offer a more sensitive correlation with PD and clinical responses. Additional sensitivity can be achieved through an orthogonal approach using multiple binding tests.

### 8.4. Pharmacokinetics

Since all responses and their time profile depend on the pharmacokinetic profile, a highly comparable PK profile should support PD similarity. The classical bioequivalence model should also work well for biological drugs. While a single-compartment model is simple to interpret, multicompartmental models can also be modeled to represent the clearance model. One additional PK parameter is the distribution volume and its rate of change [109,110], which has been suggested as a determinant of the onset of action that can be compared to add more validity to the role of PK data. The FDA also promotes using in silico pharmacokinetic studies instead of testing in patients [111].

## 9. Interchangeable Biosimilars

Interchangeability is only an issue in the US, wherein the legislation created two classes of biosimilars; the EMA and MHRA have declared that all biosimilars are interchangeable. The BPCIA creates two categories of biosimilar products: biosimilar and interchangeable biosimilar. The latter classification was intended to allow the automatic substitution of an originator product with a biosimilar product at the dispensing level. The complexity of the evaluation of interchangeable products, where the reference product and the biosimilar candidate products are switched and alternated in a patient population to demonstrate that there is no reduction in efficacy or any increase in the side effects, is more complex and less reliable than establishing comparable efficacy in the first place. While it should have been evident that removing interchangeability was necessary, this is not within the power of the FDA; however, the FDA can approve products as interchangeable without the prescribed switching and alternating studies, as the FDA has done before [112].

The FDA has significantly shifted its priorities and funding research to find alternatives to interchangeability testing and resolve issues related to analytical assessment and interaction with primary packaging materials. In October 2022, the FDA released a final guideline on using multiple endpoints in clinical testing [113] and updated the guideline on applying statistical methods to establish efficacy and comparative efficacy in establishing interchangeability [114]. However, the scientific rationale around such testing remains unresolved. This regulatory status has become a political rift as some states blocked substituting interchangeable products, and others specifically allowed this substitution [115].

The author is working with the US Senate to place a bill before the Senate to remove the interchangeable status from the BPCIA since it creates two classes [116,117], leading to a lack of confidence in biosimilars and leaving the testing options in most cases, to larger companies developing biosimilars. I am suggesting that the FDA support the bill in the Senate to amend the BPCIA to remove the interchangeable class of biosimilars.

## 10. Conclusions

It is now widely recognized that testing biosimilars in patient populations does not add to further confidence in the safety and efficacy evaluation, a conclusion that all regulatory agencies now accept. The GASK approach should be sufficient for the harmonization of clinical efficacy testing, following the path taken by MHRA. Alternates to CES, such as PD biomarkers, are just as uncertain and impractical to validate, making receptor binding and known PD biomarker comparisons a better choice to establish clinical equivalence. One reason to remove clinical efficacy testing is to avoid ethical concerns as codified in the US 21 CFR 320.25(a)(13), namely regarding the universal belief that “No unnecessary human testing should be performed”. The hazardous concerns arise from the possibility of justifying critical analytical and pharmacology profile differences based on efficacy testing in patients. If the FDA is willing to accept its advice—and it should—then the PK study alone should be sufficient to establish clinical efficacy.

Other GASK applications may include in vitro immunogenicity testing, rational analytical assessment, and accepting the monographs from the USP. Some changes will require congressional actions, such as eliminating the patent dance and interchangeable status, even though the FDA could further decide that this issue is not a quality matter, as it did when revising the labeling of biosimilars.

Like all biologics, biosimilars may elicit unwanted immune responses that can significantly impact clinical efficacy and safety. Head-to-head immunogenicity assessment of biosimilars and their reference biologics should, therefore, be a critical component of a biosimilar’s clinical development program. However, many bioanalytical platforms may be used to detect and characterize immune responses, each having relative strengths and weaknesses, requiring interpreting immunogenicity results in an assay-specific context as well as in the perspective of clinical pharmacology, efficacy, and safety [118].

Biosimilars have come a long way; the safety record of post-market analysis in the US and EU and the conclusions drawn from thousands of clinical studies have resulted in enough scientific knowledge to not only remove redundant studies but also to harmonize the biosimilar approval process across the significant jurisdictions to enable global distribution with a simpler registration dossier. The GASK Guidance may not be directly applicable to the recommendations made in this paper, but in its spirit, it completely supports the suggestions made.

The FDA has allowed the licensing of biosimilars without clinical efficacy testing [62] as well as enabling interchangeable status [112] without the switching and alternating testing; now, this concept needs to be extended to all biosimilars.

The FDA should consider the recommendations made in this paper and update the scope of the GASK Guidance to enable other propositions to come up that will remove many misconceptions and misunderstandings in the development of drugs, more specifically, biological drugs, whose cost of development as new drugs or biosimilars remains exorbitant and needs to be brought down without compromising the safety and efficacy evaluation when altered based on sound scientific principles.

## Figures and Tables

**Table 1:** Lawsuits filed (resolved) against biosimilar candidates (Effective 21 September 2023) [39].

Drug	Number of Lawsuits (#Resolved)
Aflibercept	1
Denosumab	1
Natalizumab	1
Tocilizumab	1
Adalimumab	5 (5)
Bevacizumab	4 (4)
Epoetin alfa	2 (2)
Etanercept	2 (2)
Filgrastim	5 (5)
Infliximab	2 (2)
Pegfilgrastim	6 (6)
Rituximab	2 (2)
Trastuzumab	5 (5)
Ustekinumab	1 (1)

**Table 2:** PD markers for all FDA-licensed monoclonal antibody types.

Therapeutic Protein Class	PD Marker	Receptor Binding Test
Anti-CD20 antibodies (e.g., Rituximab) [75]	CD20+ B-cell counts, immunoglobulin levels	CD20 binding assay
Anti-IgE therapies (e.g., Omalizumab) [76]	Serum-free IgE levels, basophil activation	IgE binding assay
Anti-IL-5 therapies (e.g., Mepolizumab, Reslizumab) [77]	Blood eosinophil count, asthma exacerbation rates	IL-5 receptor binding assay
Anti-PD-1 antibodies (e.g., Nivolumab, Pembrolizumab) [78]	Tumor responses, immune cell activation	PD-1 binding assay
Anti-VEGF agents (e.g., Bevacizumab, Aflibercept) [79]	Tumor response, normalization of tumor vasculature	VEGF binding assay
Bruton’s tyrosine kinase (BTK) inhibitors (e.g., Ibrutinib) [80]	B-cell counts, lymph node size, BTK phosphorylation	BTK binding assay
BTK inhibitors (e.g., Ibrutinib) [81]	B-cell counts, lymph node size, BTK phosphorylation	BTK binding assay
CD20-directed therapies (e.g., Rituximab) [82]	B-cell counts, tumor response in B-cell malignancies	CD20 binding assay
CD3-directed therapies (e.g., Blinatumomab) [83]	T-cell counts, tumor response in B-ALL	CD3 binding assay
CD38-directed therapies (e.g., Daratumumab) [84]	Plasma cell counts in multiple myeloma, immunoglobulin levels	CD38 binding assay
Coagulation factors (e.g., Factor VIII, Factor IX) [85]	Clotting times, bleeding episodes	N/A (Functional activity assays used)
Complement C5 inhibitors (e.g., Eculizumab) [86]	Hemolysis markers, renal function in aHUS	Complement C5 binding assay
CTLA-4 inhibitors (e.g., Ipilimumab) [87]	Tumor response in melanoma, immune cell activation	CTLA-4 binding assay
EGFR inhibitors (e.g., Cetuximab, Panitumumab) [88]	Tumor response, skin rash, EGFR phosphorylation levels	EGFR binding assay
Factor IX products (e.g., BeneFIX) [89]	Control and prevention of bleeding episodes in Hemophilia B	Factor IX binding assays
Factor VIII products (e.g., Advate) [90]	Control and prevention of bleeding episodes in Hemophilia A	Factor VIII binding assays
Factor Xa inhibitors (e.g., Andexanet alfa) [91]	Bleeding control, clotting times	Factor Xa binding assay
HER2/neu-directed therapies (e.g., Trastuzumab) [92]	Tumor response, cardiac monitoring, HER2 phosphorylation	HER2/neu binding assay
IL-1 inhibitors (e.g., Anakinra, Canakinumab) [93]	Inflammatory markers, clinical scores	IL-1 receptor binding assay
IL-1 inhibitors (e.g., Anakinra) [94]	Symptom relief in RA, systemic JIA; inflammatory markers	IL-1 receptor binding assay
IL-12/23 inhibitors (e.g., Ustekinumab) [95]	Cytokine levels (IL-12, IL-23), PASI score in psoriasis	IL-12/IL-23 p40 subunit binding assay
IL-17 inhibitors (e.g., Secukinumab, Ixekizumab) [96]	PASI score in psoriasis, inflammatory markers in spondylitis	IL-17 receptor binding assay
IL-23 inhibitors (e.g., Guselkumab) [97]	PASI score in psoriasis	IL-23 receptor binding assay
IL-5 inhibitors (e.g., Mepolizumab) [98]	Eosinophil counts, symptom control in severe asthma	IL-5 receptor binding assay
IL-6 inhibitors (e.g., Tocilizumab) [99]	CRP, IL-6 serum levels, clinical scores in diseases like RA	IL-6R binding assay
JAK inhibitors (e.g., Tofacitinib, Baricitinib) [98]	Inflammatory markers, clinical scores	JAK protein binding assay (biochemical assay)
mTOR inhibitors (e.g., Everolimus) [99]	Tumor response, organ transplant graft survival	mTOR binding assay
PCSK9 inhibitors (e.g., Evolocumab, Alirocumab) [100]	Serum LDL cholesterol levels	PCSK9 binding assay
PD-1 inhibitors (e.g., Pembrolizumab) [101]	Tumor response in various cancers	PD-1 receptor binding assay
PD-L1 inhibitors (e.g., Atezolizumab) [102]	Tumor responses, immune cell activation	PD-L1 binding assay
SGLT2 inhibitors (e.g., Dapagliflozin, Empagliflozin) [103]	Blood glucose levels, HbA1c levels	SGLT2 receptor binding assay
Soluble guanylate cyclase (sGC) stimulators [104]	Plasma cyclic GMP levels, vasodilatory responses	sGC binding assay
TNF-α inhibitors (e.g., Infliximab, Adalimumab) [105]	Inflammatory markers (CRP, ESR), clinical scores in diseases like RA	TNF-α binding assay

## Data Availability

Data is contained within the article.
